# Liver metastasis from colorectal cancer: pathogenetic development, immune landscape of the tumour microenvironment and therapeutic approaches

**DOI:** 10.1186/s13046-023-02729-7

**Published:** 2023-07-22

**Authors:** Yaxian Wang, Xinyang Zhong, Xuefeng He, Zijuan Hu, Huixia Huang, Jiayu Chen, Keji Chen, Senlin Zhao, Ping Wei, Dawei Li

**Affiliations:** 1 Department of Colorectal Surgery, Fudan University Shanghai Cancer Center, Shanghai, China; 2 Department of Oncology, Shanghai Medical College of Fudan University, Shanghai, China; 3 ZJU-UCLA Joint Center for Medical Education and Research, Cancer Institute, The Second Affiliated Hospital, Zhejiang University School of Medicine, Hangzhou, China; 4 Department of Pathology, Fudan University Shanghai Cancer Center, Shanghai, China; 5 Cancer Institute, Fudan University Shanghai Cancer Center, Shanghai, China; 6 Institute of Pathology, Fudan University, Shanghai, China

**Keywords:** Colorectal cancer liver metastasis, Premetastatic niche, Immune landscape, Tumour microenvironment, Immunotherapy

## Abstract

Colorectal cancer liver metastasis (CRLM) is one of the leading causes of death among patients with colorectal cancer (CRC). Although immunotherapy has demonstrated encouraging outcomes in CRC, its benefits are minimal in CRLM. The complex immune landscape of the hepatic tumour microenvironment is essential for the development of a premetastatic niche and for the colonisation and metastasis of CRC cells; thus, an in-depth understanding of these mechanisms can provide effective immunotherapeutic targets for CRLM. This review summarises recent studies on the immune landscape of the tumour microenvironment of CRLM and highlights therapeutic prospects for targeting the suppressive immune microenvironment of CRLM.

## Introduction

Colorectal cancer (CRC) is the third most common cancer and the second leading cause of cancer-related mortality worldwide [1]. Although colonoscopy screening has become popularised, the morbidity and mortality of CRC remain high among men [2]. Early-stage CRC is eligible for curative treatment [3]; however, 25–50% of patients with early-stage disease progress to metastatic disease [4]. The liver is the most frequent site of metastasis in patients with CRC [5]. Blood draining from the gastrointestinal tract enters the liver through the portal vein, which promotes the dissemination of CRC into the liver [6, 7]. Approximately 15–25% of patients with CRC have synchronous liver metastasis (LM) [8, 9], and 18–25% of patients with CRC may eventually develop metachronous LM within 5 years of the initial diagnosis [10]. The 5-year survival rate dramatically declines when the local disease develops into metastasis [11, 12]. Therefore, LM has been used as a prognostic marker for CRC. Despite the development of surgical techniques and targeted therapy, the prognosis of colorectal liver metastasis (CRLM) remains poor [13].

The tumour microenvironment (TME) is composed of cancerous and noncancerous cells, including fibroblasts, endothelial cells and immune cells, as well as noncellular components such as the extracellular matrix (ECM), cytokines, growth factors and extracellular vesicles (EVs) [14, 15]. The immune landscape of the TME is intrinsically correlated with the progression and metastasis of cancer [16, 17]. Immunosuppressive cells mediate suppressive immune activities against effector lymphocytes, thus leading to the formation of an immunosuppressive TME [18–20]. To adapt to various antigens from the gut, the liver performs unique immunoregulatory functions, which are mainly determined by antigen-presenting cells (APCs) with tolerogenic capabilities to maintain immune system homeostasis [21–23]. In addition, resident cells in the liver play a critical role in the invasion of CRC by interacting with metastatic CRC cells. The TME of LM has a highly immunosuppressive phenotype, which is indicated by the loss of antigen-specific CD8^+^ T cells; thus, this TME promotes the invasive and metastatic capabilities of primary cancer cells [24, 25]. Due to the fact that the immune landscape of the TME is associated with the response to immunotherapy, a better understanding of the immune landscape of the TME in CRLM may help to manage patients with LM.

In this review, we described the pathogenetic development of CRLM and the immune landscape of the TME in CRLM and discussed various therapies for CRLM. In addition, we highlighted possible approaches for targeting the immune microenvironment to open new immune–oncology avenues that will promote future research.

## Liver metastasis from colorectal cancer

### Hepatotropism of cancer metastasis

Upon encountering a unique organ microenvironment, disseminated cancer cells exhibit site-specific tropism to help themselves adapt and survive [26, 27], which is also described as possessing a high affinity for certain organs. After its implantation into compatible ‘soil’, the ‘seed’ can easily colonise and grow. Numerous studies have demonstrated the mechanisms that drive the metastasis of primary cancer to specific organs in a manner that cannot be solely explained by circulatory patterns [28–36]. Solid malignancies exhibit unique and recurrent organ tropism to specific secondary sites, including the liver, lung, bones and pleura [37].

**Fig. 1:**
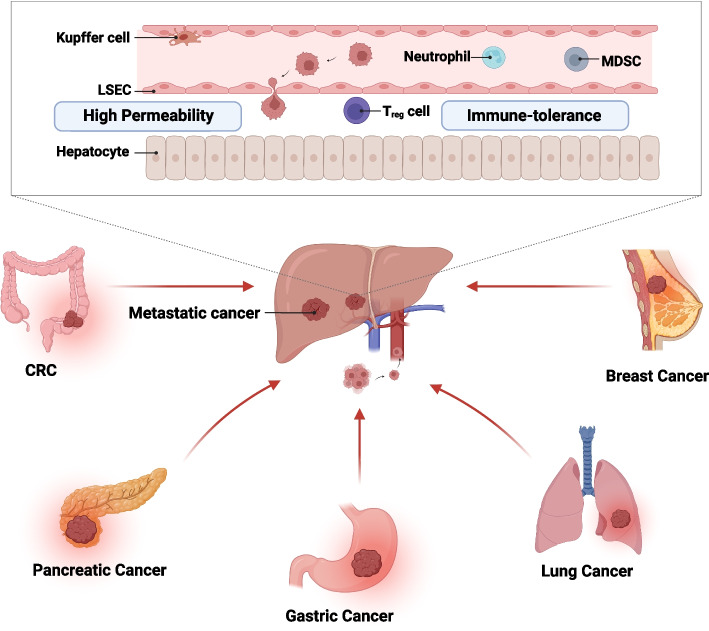
Hepatotropism of cancer metastasis to liver

As an immunological organ and central metabolic organ, the liver is a highly metastasis-compatible organ that can be colonised by multiple primary cancers, including CRC, pancreatic cancer, gastric cancer, lung cancer, breast cancer and melanoma [38–40]. The incidence rate of LM has been reported to be higher than that of primary liver cancer [41]. Moreover, the unique structure and following characteristics of the liver make it intrinsically susceptible to bloodborne metastasis. (1) The dual blood supply through the hepatic portal vein and hepatic artery provides more chances for circulating cancer cells to invade the liver. This phenomenon underlies the development of most metastases from primary cancer to specific secondary organs [42, 43]. (2) The slow blood flow and high permeability of fenestrated liver sinusoidal endothelial cells (LSECs) promote the invasive capability of disseminated cancer cells [44, 45]. (3) The immune-tolerance ability of the liver shapes the immunosuppressive microenvironment, which prevents damage caused by overreactions to antigens entering the liver [21–23, 46] (Fig. 1).

The liver is the most popular site for the metastasis of CRC [47]. The rich and slow portal venous supply from the gut to the liver and the immune-tolerance ability of the liver can partly explain the frequency of CRLM [38]. The right side (especially the hepatic flexure of the colon) is adjacent to the liver, which can cause the direct spread of CRC into the liver [48]. Additionally, the expression of chemokines on CRC cells is responsible for liver-specific metastasis [49]. The high expression of CXCL12 in the liver delivers specific homing signals for CRC cells that have a high expression of CXCR4 receptors, thus contributing to liver-specific metastasis in CRC [50]. Furthermore, the CCR6–CCL20 signalling pathway between CRC and the liver is independently implicated in the occurrence of CRLM [51]. Altogether, the liver represents a fertile ‘soil’ for circulating CRC cells (‘seeds’) to spread and grow.

### Pathogenetic development of CRLM

The pathogenetic development of CRLM is mainly divided into four overlapping phases [47, 52, 53]. (1) Microvascular phase: Liver-infiltrating CRC cells that are trapped in sinusoidal vessels are killed via phagocytosis by Kupffer cells (KCs) and natural killer (NK) cell-mediated antitumour cytotoxicity [54, 55]; they may also remain alive by escaping cytotoxic effects and adhering to LSECs [53], which facilitates CRC migration into the space of Disse to avoid immune killing. (2) Extravasation and preangiogenic phase: CRC cells relocate to the space of Disse, thus recruiting stromal cells, including hepatic stellate cells (HSCs) that are responsible for the secretion of fibronectin and collagen to form a framework for neovascularization [56, 57] and portal tract fibroblasts, which generate IL-8 to promote invasion and angiogenesis [58]. (3) Angiogenic phase: After LSECs are activated and co-opted to the tumour–liver interface, activated HSC-derived vascular endothelial growth factor (VEGF) induces the formation of intrametastatic vessels, which appear to be continuous with sinusoidal vessels [59]. Various immunosuppressive cells, such as immunosuppressive regulatory T (T_reg_) cells, myeloid-derived suppressor cells (MDSCs) and macrophages, are activated to form an immunosuppressive microenvironment, which promotes the development of CRLM. (4) Growth phase: CRC cells acquire adequate blood supply and proliferate rapidly under the ‘protection’ of intrinsic hepatic immune tolerance and the immunosuppressive microenvironment, eventually forming a detectable metastatic tumour in clinical settings [47]. Therefore, the targeting of angiogenesis and the transformation of the immunosuppressive microenvironment into an immune-effective microenvironment are prospective therapeutic strategies for CRLM. Moreover, an understanding of the immune microenvironment of the liver may help to develop effective immunotherapeutic approaches.

## Immune landscape of the TME in CRLM

**Fig. 2:**
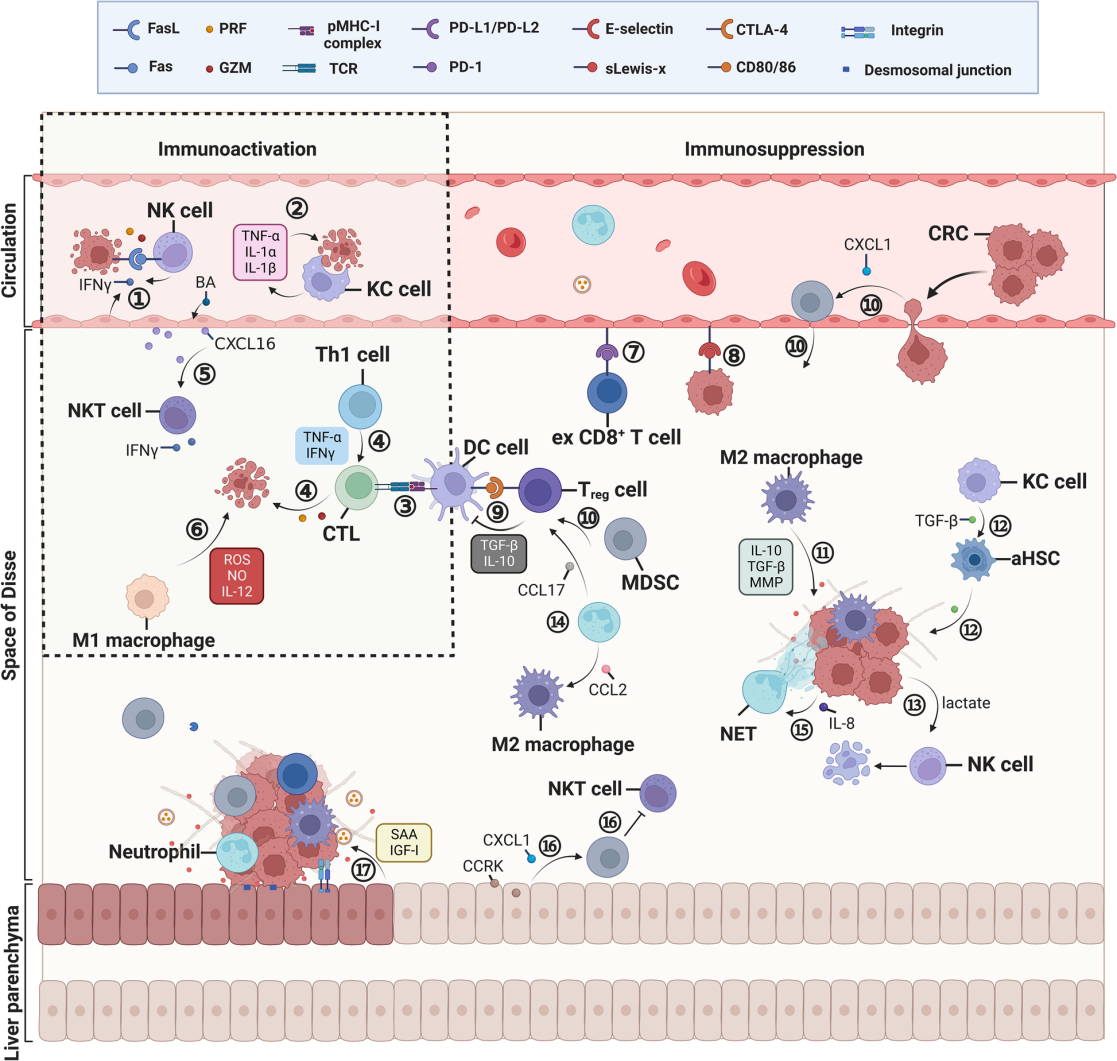
A schematic representation of the immune landscape of the TME in CRLM. GZM, granzyme; PRF, perforin; CTL, cytotoxic T lymphocyte; BA, bile acid; ex CD8^+^ T cell, exhausted CD8^+^ T cell; NET, neutrophil extracellular trap; CCRK, cell cycle-related kinase; sLewis-x, sialyl Lewis-x; CTLA-4, cytotoxic T lymphocyte antigen-4; SAA, serum amyloid A1 and A2; IGF-I, insulin-like growth factor-I. ① LSECs and NK cells produce IFNγ to upregulate functional Fas and induce apoptosis of cancer cells; PRF and GZM released from NK cells kill cancer cells. ② Disseminated CRC cells are phagocytosed by KCs along with the release of TNF-α, IL-1α and IL-1β. ③ APCs present neoantigens to CD8^+^ T cells, thus inducing the rapid proliferation of CD8^+^ T cells and their differentiation into CTLs. ④ CTLs secrete PRF and GZM, as assisted by IFNγ and TNF-α produced by Th1 cells to kill cancer cells. ⑤ LSECs are regulated by gut microbiota-modified bile acids to secret CXCL16, thus recruiting NKT cells to fight cancer cells. ⑥ M1 macrophages directly kill cancer cells by releasing cytotoxic ROS, NO and IL-12. ⑦ The function of cytotoxic CD8^+^ T cells is impeded due to the interplay between PD-L1 and PD-1. ⑧ The interaction between E-selectin and sialyl Lewis-x promotes the adhesion of CRC cells to LSECs. ⑨ Treg cells bind to APCs via the interaction between CTLA-4 and CD80/86 and produce TGF-β and IL-10 to suppress the activation of CTLs. ⑩ MDSCs, which are recruited by CXCL1 secreted from CRC cells, induce the activation of T_reg_ cells to impair the antigen-presenting activity of DC cells. ⑪ M2 macrophages produce IL-10, TGF-β and MMP to regulate matrix remodelling. ⑫ As induced by TGF-β secreted from KCs, HSCs are transformed to aHSCs and release TGF-β to promote ECM remodelling. ⑬ Lactic acid causes NK cell apoptosis by downregulating their intracellular pH. ⑭ TANs release CCL2 and CCL17 to recruit CCR2^+^ M2 macrophages and CCR4^+^ Treg cells. ⑮ As induced by IL-8, NETs trap CRC cells in the liver. ⑯ Hepatocyte-derived CCRK increases CXCL1 production to recruit PMN-MDSCs, thereby impairing NKT cell-mediated immunosurveillance. ⑰ As mediated by integrins and desmosomes, CRC cells adhere to hepatocytes, thus inducing the release of SAA and IGF-I from hepatocytes

The homeostasis maintained by organ innate resistance in the liver is attributed to various highly specialised resident cells and all types of immune cells [21, 60–62]. Each of these cells not only helps to balance protein, lipid and glucose metabolism but also orchestrates immune responses and oncogenesis [63–68]. The considerable inflow of antigens shapes the unique immune microenvironment of the liver to harmonise immune activation and immune tolerance [69, 70]. In the early stage of CRLM, the abovementioned cells act as defenders to destroy disseminated cancer cells. Specifically, LSECs arrest cancer cells, whereas KCs phagocytose and release tumour-killing cytokines. Additionally, APCs present antigens to T cells and transform them to effector T cells, which is strengthened by CD4^+^ T cells. Cytokines released from natural killer T (NKT) cells and M1 macrophages protect against cancer cells. However, when cancer cells escape the immune system, effector T cells are rendered dysfunctional by immune checkpoints, whereas cancer cells migrate into the space of Disse by adhering to LSECs. Treg cells impair the antigen-presenting activity of dendritic cells (DCs). Moreover, HSCs are activated to promote ECM remodelling, and M2 macrophages produce MMPs to regulate this process. Tumour-associated neutrophils (TANs) extrude chromatin fibres and form neutrophil extracellular traps (NETs), which trap CRC cells in the liver and eventually promote their invasive and metastatic capabilities. Furthermore, CRC cells adhere to hepatocytes and induce the release of serum amyloid A1 and A2 (SAA) and insulin-like growth factor-I (IGF-I) from hepatocytes, thereby making the liver a primary target for CRC metastasis (Fig. 2).

### Interaction of resident liver cells with cancer cells

#### Liver sinusoidal endothelial cells

LSECs perform important physiological and immunological functions, including filtration, endocytosis and antigen presentation [71–73]. As a selective barrier, LSECs allow for the entry of molecules such as plasma proteins, drugs, small chylomicron remnants, exosomes and smaller viruses (< 200 nm) into the space of Disse; however, they do not allow the entry of cells [74–76]. The mannose receptor, scavenger receptor and Fc-γ receptor IIb2 efficiently facilitate the clearance and degradation of blood-borne macromolecules by LSECs to perform endocytosis and scavenging functions [77, 78]. Additionally, LSECs exert antigen-presenting functions mediated by the mannose receptor and scavenger receptor, which mainly reshape the immunosuppressive microenvironment in the liver. However, LSECs can dampen effector immune responses. Specifically,, antigen presentation by LSECs mainly induces the differentiation of CD4^+^ T cells into T_reg_ cells to promote the development of an immune-tolerant TME in the liver [44, 79]. In contrast, LSECs mainly drive a tolerogenic response mediated by an increase in the levels of coinhibitory PD-L1 that interacts with PD-1 to induce CD8^+^ T-cell dysfunction [80].

LSECs play a dual role in advancing tumorigenesis. When disseminated CRC cells enter the sinusoids, they are entrapped by LSECs and are either destroyed due to mechanical stress, phagocytosed by KCs or killed by perforin (PRF)/granzyme (GZM) from NK cells. LSECs and NK cells release interferon-gamma (IFNγ) and nitric oxide (NO) to upregulate Fas and induce apoptosis of cancer cells via the Fas–FasL pathway [81]. Recent studies have highlighted the fact that LSECs are influenced by gut microbiota-modified bile acids to secrete CXCL16, which recruits NKT cells to fight primary and metastatic liver tumours [82]. However, the anticancer proinflammatory response results in the high expression of vascular adhesion factors such as E-selectin, VCAM-1 and ICAM-1 on LSECs, thus leading to the susceptibility of LSECs to adhesion by cancer cells with the help of sialyl Lewis-x, PSGL-1 and ESL-1 [83–85]. Cancer cells can escape from the destruction of the initial assault through counterreceptor communication, after which they migrate into the space of Disse, where they are protected from the cytotoxic effects of KCs and NK cells [86]. A novel adhesion molecule known as LSECtin mediates the communication between activated T cells and LSECs [87, 88] to inhibit the tumour-killing effects of T cells; in addition, it facilitates adhesion and migration of CRC cells to the liver [89]. In multiple experimental LM models, melittin nanoparticles have been demonstrated to induce the activation of LSECs to reverse the hepatic immunological environment to the activated state, which recruits NK and CD8^+^ T cells and suppresses LM [90].

Due to the fact that LSECs induce a suppressive immune microenvironment in the liver and assist in the growth of disseminated cancer cells, the targeting of LSECs to modulate the hepatic immune microenvironment may be a novel approach to the management of LM in the future. For example, the abundance of beneficial gut organisms that optimise the metabolism and immunity of the liver can be enhanced by modulating the action of LSECs for the effective treatment of CRLM.

#### Kupffer cells

KCs, which are the resident macrophages in the liver, serve as a crucial part of the innate immune response, which is the first line of defence of the liver [91]. Localised in the hepatic sinusoid, KCs can recognise all types of antigens (such as immune complexes, senescent cells and cancer cells) from the portal or arterial circulation and exert anti-inflammatory effects to prevent the entry of gut-derived substances into the hepatic sinusoid [92, 93]. In the early stage of CRLM, the adherence of disseminated cancer cells to KCs prompts KCs to capture and phagocytose the cancer cells and release TNF-α, interleukin-1α (IL-1α) and IL-1β, thus reducing the metastasis of colon cancer cells to the liver [94, 95]. The innate receptor Dectin-2 on KCs promotes the phagocytosis and elimination of disseminated CRC cells to resist metastasis [96].

Although KCs mainly play a tumoricidal role in the early stages of metastasis, they also play a vital role in hepatic carcinogenesis [97]. KCs activate and expand FOXP3^+^CD4^+^ T_reg_ cells through antigen presentation and induce tolerance by upregulating the inhibitory marker PD-L1, thereby resulting in the formation of an immune-tolerant environment to achieve homeostasis [23]. Moreover, HSCs are activated and produce fibronectin induced by KC-derived TGF-β, thus recruiting bone marrow-derived macrophages and neutrophils to form a favourable environment [53]. The corelease of TGF-β, fibronectin, EGF, VEGF and matrix metalloproteinases (MMP-2, MMP-9 and MMP-13) from KCs and HSCs leads to ECM remodelling, angiogenesis and cancer progression [98], which is augmented by the absorption of pancreatic ductal adenocarcinoma (PDAC)-derived exosomes by KCs in a PDAC model [99]. However, CRC-derived exosomal angiopoietin-like protein 1 (ANGPTL1) shuttles to KCs to decrease the expression of MMP-9, which subsequently reduces LM and inhibits vascular leakage mediated via the suppression of the JAK2-STAT3 signalling pathway [100]. In addition, KCs can phagocytose EV-packaged miR-135a-5p, thus mediating immunosuppression and facilitating the development of a premetastatic niche (PMN) in patients with CRLM [101].

It has been reported that a novel immunotherapy strategy by using bacterial genetic modification induces the reprogramming of KCs, which augments the phagocytic ability of cancer cells and strengthens the cytotoxic killing capacity of T cells to suppress LM [102]. An understanding of the functional role of KCs in CRLM may help to identify potential therapeutic targets and to develop novel therapeutic strategies, such as nanoparticle-mediated noncoding RNA-based therapy and bacterial treatment to reprogram the function of KCs. However, further research is required to identify the underlying mechanism and potential for application.

#### Hepatic stellate cells

As a resident nonparenchymal liver cell population, HSCs contribute to liver fibrosis and cancer development [67, 103]. HSCs maintain homeostasis in the liver by regulating the ECM, immune tolerance and inflammatory responses; additionally, they play a significant role in the colonisation and metastasis of cancer cells [104–106].

TGF-β is an important regulator of HSCs in the hepatic microenvironment. Its high expression blocks the initiation of CD4^+^ Th1 cells and weakens cytotoxic responses, thus facilitating LM and leading to a poor prognosis [107, 108]. TGF-β can induce the transformation of HSCs into a fibroblast-like (spindle-like and spread) phenotype (known as activated HSCs [aHSCs]) to promote ECM remodelling [98]. In addition, aHSCs play a vital role in secondary or primary hepatocellular carcinoma [109–111]. aHSCs can lead to hepatic fibrosis and portal hypertension, thus contributing to hepatocarcinogenesis and metastasis [109]. In a previous study, we demonstrated that CRC-derived exosomal miR-181a-5p facilitates CRLM by activating HSCs [112]. In addition, aHSCs engulf disease-associated lymphocytes, including CD8^+^ T, CD4^+^ T and NK cells, through cell adhesion [113]. As key cells involved in pro-tumour angiogenesis, HSCs have been demonstrated to upregulate fibroblast activation protein alpha (FAPα) and increase CXCL5 secretion, as regulated by cancer cell-secreted fibroblast growth factor-binding protein 1 (FGFBP1). This mechanism stimulates epithelial–mesenchymal transition (EMT) and induces vessel co-option that results in bevacizumab resistance in CRLM models [114]. Therefore, HSCs play an important role in shaping the immune microenvironment of the liver and in inducing resistance to antiangiogenic therapy. The targeting of HSCs expressing specific molecules (such as FAPα) to modulate the immune microenvironment of CRLM represents a beneficial strategy for strengthening the antitumour effects of immune cells and for effectively overcoming drug resistance.

#### Hepatocytes

Hepatocytes play an essential role in inducing an immune-tolerant TME, which is required for the implantation of disseminated cancer cells. Hepatocyte-mediated cross-presentation of soluble antigens can induce tolerance of antigen-specific CD8^+^ T cells [115]. After extravasation, disseminated CRC cells can deeply penetrate into the hepatocyte plate, where they proliferate and form metastatic foci. The adhesion of CRC cells to hepatocytes is considered an essential step in the formation of LM [116], which is mediated by integrins [116] or desmosomes [117]. The strongly expressed integrin subunit αvβ5 mediates cell migration and LM in CRC, and its effects are enhanced by hepatocyte-derived heregulin [118]. Hepatocyte-derived SAA can facilitate the development of LM and is highly expressed in patients with CRC. Mechanistically, hepatocytes promote LM by activating IL-6–STAT3 signalling and inducing SAA overexpression, thereby reshaping the hepatic TME to facilitate the formation of a PMN in the liver [119]. Moreover, IGF-I can affect cancer growth and metastasis. The inhibition of IGF-1 released from hepatocytes reduces CRLM in mice [120]. A novel IGF-targeting protein (IGF-Trap) has been demonstrated to markedly block CRLM in experimental models to compensate for the function of the impaired insulin receptor system, thus inducing tumour cell apoptosis and reducing angiogenesis [121].

Altogether, the interplay between resident liver cells and cancer cells contributes to the progression and spread of CRC (Table 1). A better understanding of the communication between CRC cells and the hepatic TME may facilitate the development of new combination therapies for the efficient management of CRLM.

**Table 1:** Interaction between resident liver cells and cancer cells

Resident cells	Interacting molecule(s)	Major effects
LSECs	Mannose receptor and scavenger receptor	Internalise, process and transfer antigens through MHC I and MHC II to T cells
	PD-L1–PD-1	Induce CD8^+^ T-cell tolerance to trigger immune escape of cancer cells
	IFNγ, NO and Fas–FasL	Induce apoptosis of cancer cells
	CXCL16	Recruit NKT cells to fight cancer cells
	E-selectin, VCAM-1, ICAM-1, sialyl Lewis-x, PSGL-1 and ESL-1	Facilitate the adherence of cancer cells to LSECs and their migration into the space of Disse to protect them from elimination
	LSECtin	Suppress T-cell immune responses and promote the adhesion and metastasis of CRC to the liver
KCs	TNF-α, IL-1α and IL-1β	Phagocytose and eliminate disseminated cancer cells
	MHC II and PD-L1–PD-1	Expand T_reg_ cells to induce an immune-tolerant environment
	TGF-β, fibronectin, EGF, VEGF, MMP-2, MMP-9 and MMP-13	Lead to ECM remodelling, angiogenesis and cancer progression
	Exosomal miR-135a-5p	Mediate immunosuppression and facilitate the formation of a pre-metastatic niche
HSCs	TGF-β, fibronectin, EGF, VEGF, MMP-2, MMP-9, and MMP-13	Lead to ECM remodelling, angiogenesis and cancer progression
	TGF-β	Promote ECM remodelling
	Exosomal miR-181a-5p	Facilitate CRLM by activating HSCs
Hepatocytes	Integrins or desmosomes	Mediate the adhesion of CRC cells to hepatocytes
	Integrins and heregulin	Boost the migration and LM of CRC
	SAA	Reshape the hepatic immune and fibrogenic microenvironment to promote LM
	IGF-1	Promote cancer growth and metastasis

*MHC I *Major histocompatibility complex class I, *MHC II *Major histocompatibility complex class II, *SAA *Serum amyloid A1 and A2,  *IGF-1 *Insulin-like growth factor-I

### Immune cells contributing to the hepatic immune microenvironment of CRLM

#### CD4^+^ T cells

CD4^+^ T cells are essential in the defence against tumours because they regulate the activity of CD8^+^ T cells and influence the outcome of antitumour responses [122]. The classical effector CD4^+^ T helper 1 (Th1) and T helper 2 (Th2) subsets elicit important antitumour immune responses. Specifically, Th1 cells produce cytokines such as IFNγ and TNF-α, thus leading to cell-mediated killing, whereas Th2 cells secrete IL-4, which assists in the activation of humoral immunity [123]. In addition, CD4^+^ T cells can differentiate into new subsets, such as Th9 cells, Th17 cells and FOXP3^+^ T_reg_ cells. Moreover, the role of Th17 cells in cancer is controversial [124, 125]. The low proportion of Th1 cells and high proportion of Th17 cells in liver metastatic tissue indicate a poor prognosis in patients with CRLM [126], which is consistent with the condition of patients with CRC [127]. Given the immunosuppressive activity of CD4^+^ T cells, we mainly focused on FOXP3^+^CD4^+^ T_reg_ cells in this review.

One of the characteristics of LM progression is the high infiltration of FOXP3^+^ T_reg_ cells [128]. The expression of PD-1 on T_reg_ cells is higher in highly glycolytic LM tissue than in primary cancer tissue; however, it is lower in CD8^+^ T cells, which contributes to resistance to anti-PD-1 treatment [129]. The enrichment of T_reg_ cells in the TME is responsible for cancer immune evasion [130], which can partly explain the worse prognosis of CRLM. The immunosuppressive mechanism of action of T_reg_ cells occurs in the following way. (1) Interaction with APCs: Compared with CD28 expressed by naïve T cells, cytotoxic T lymphocyte antigen-4 (CTLA-4) expressed by activated Treg cells has a higher affinity for CD80/86 found on APCs [131, 132]. (2) The use of immune-suppressive metabolites: T_reg_ cells metabolise ATP to adenosine through CD39 and CD73, and the adenosine–A_2A_ receptor (A_2A_R) interaction inhibits effector T cells [133]. (3) Involvement of cytokines: T_reg_ cells produce high amounts of CD25 by using IL-2, thus leading to the availability of a low level of IL-2 for activating effector T cells. Additionally, the high levels of TGF-β, IL-10 and IL-35 released from T_reg_ cells inhibit the activation of effector T cells [134–136]. Moreover, TGF-β mediates EMT to promote the metastasis of disseminated CRC cells [137, 138]. Therefore, the elimination of the suppressive TME induced by T_reg_ cells may be a beneficial approach to reviving effector antitumour responses.

#### CD8^+^ T cells

CD8^+^ T cells contribute to the clearance of intracellular pathogens and malignant cells and support long-term protective immunity [139, 140]. Based on distinct immune profiles, CD8^+^ T cells can be classified as exhausted (ex) CD8^+^ T cells, effector CD8^+^ T cells (which are also known as cytotoxic T lymphocytes [CTLs]) and memory CD8^+^ T cells [141]. CD8^+^ T cells serve as a useful marker to predict prognosis and therapeutic efficacy in cancer [142].

Upon encountering cancer cells, CD8^+^ T cells are activated by TCR-recognised antigens and rapidly proliferate and differentiate into CTLs to eliminate cancer cells through cell-to-cell contact. After CTLs are conjugated to target cancer cells, they secrete cytotoxic granules and release a cargo of deadly proteins, including PRF, GZM and granulysin, to kill the target cells [143]. A high proportion of CTLs contributes to improved outcomes in CRC [144]. However, to maintain hepatic immune tolerance, effector T cells are induced to undergo anergy, differentiation or apoptosis [145, 146].

**Fig. 3:**
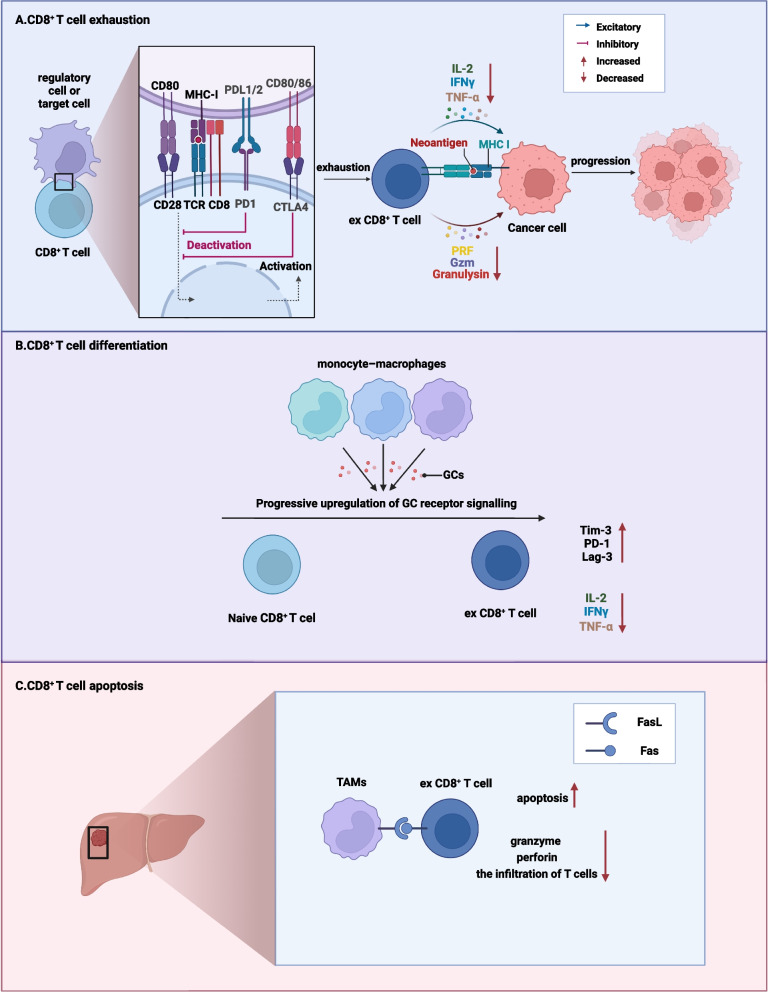
The role of CD8^+^ T cells in hepatic immune tolerance. To maintain homeostasis in the liver, which is exposed to an increased burden of harmless dietary factors and antigens, effector CD8^+^ T cells are induced to undergo anergy, differentiation or apoptosis

CTLs undergoing persistent exposure to cancer antigen signals will gradually lead to the transformation of CD8^+^ T cells to a dysfunctional state, which are known as ex CD8^+^ T cells [147]. Moreover, ex CD8^+^ T cells secrete fewer effective cytokines, including TNF-α, IL-2 and IFNγ [148], thus resulting in failure to induce efficient adaptive tumour-killing effects on disseminated CRC cells. A key hallmark of exhaustion is the high level of inhibitory cell surface receptors, including PD-1 and CTLA-4 [149, 150]. CTLA4^+^CD8^+^ T cells are relatively enriched in primary CRC and LM tissues [151]. Additionally, the high proportion of immunosuppressive regulatory cells in LM, including M2 macrophages, neutrophils and T_reg_ cells [151], directly or indirectly promotes CD8^+^ T-cell exhaustion [149] (Fig. 3A).

It has been reported that CD8^+^ T cells mediate differentiation into Tim3^+^PD1^+^CD8^+^ T cells by glucocorticoid (GC) signalling in an MC38 colon carcinoma model [152] (Fig. 3B). The progressive upregulation of GC receptor signalling from naïve CD8^+^ T cells to ex CD8^+^ T cells communicates with monocyte–macrophage lineage cells, which impairs the production of immune-effective cytokines, including IL-2, TNF-α and IFN-γ, and promotes the high expression of immunosuppressive checkpoints (such as Tim-3, PD-1 and Lag-3) in CTLs, thus shaping an immunosuppressive TME [152]. Moreover, exosomal circCCAR1 expressed by cancer cells communicates with CD8^+^ T cells to impede the degradation of PD1, thus promoting the exhaustion of CD8^+^ T cells in the liver [153]. In addition, the high expression of MGP in cancer cells from both the primary CRC or LM sites increases intracellular Ca^2+^ to boost NF-κB phosphorylation, which mediates PD-L1 upregulation in CRC cells, thus promoting CD8^+^ T-cell dysfunction [154]. Furthermore, the induction of T cells to differentiate into regulatory cells is mediated by IL-10 release from LSECs, which are prone to activating the regulatory pathway of CD4^+^ T cells to FOXP3^+^CD4^+^ T_reg_ cells [145].

Recent studies on the apoptosis of CD8^+^ T cells have demonstrated that tumour-associated macrophages (TAMs) induce apoptosis of CD8^+^ T cells and impair cytotoxic functions by reducing the expression of granzyme B and perforin in the liver [155]. The mechanism for this effect involves the fact that activated CD8^+^ T cells experience apoptotic cell death by the Fas–FasL pathway, as mediated by TAMs within the liver, which induces a decrease in activated T cells and transforms the hepatic immune microenvironment in CRLM [25] (Fig. 3C).

Given that cancer-reactive CTLs play a central role in cancer immunity, it is important to reactivate CD8^+^ T cells to suppress the progression and metastasis of CRC. It has been reported that hyper-IL-15, IL-15 and the sushi domain of the IL-15 receptor α chain augment the cytotoxic functions of CD8^+^ T and NK cells, which may be a prospective therapy to reactivate CD8^+^ T cells and recover their anticancer ability to manage CRLM [156].

#### Tumour-associated macrophages

As multifunctional APCs, macrophages are critical mediators of tumour immunity [157]. Macrophages present exogenous antigens to T cells through MHC-I and MHC-II aided by costimulatory signals, inhibitory signals or other cytokine signals to regulate T-cell activation [158]. Macrophages that infiltrate malignant tissues are known as TAMs.

With inherent plasticity and polarising characteristics, TAMs are conventionally categorised into two subtypes: M1 and M2 macrophages [159, 160]. M1 macrophages suppress cancer growth by releasing cytotoxic reactive oxygen species (ROS), NO and IL-12, which can directly kill cancer cells [161]. However, M2 macrophages induce the formation of an immunosuppressive TME by secreting cytokines, including IL-10, TGF-β, CCL17 and CCL22 [157, 162]. Due to their poor ability to present cancer antigens, M2 macrophages undermine Th1 adaptive immunity [163]. In addition, M2 macrophages produce MMPs to regulate matrix remodelling, thus facilitating the invasion and metastasis of cancer [164]. In CRC, the expanding liver metastatic tumour is rich in TAMs (primarily M2 macrophages) [157, 165], which are recruited through the CCL2/CCR2 chemokine axis to form an immunosuppressive microenvironment [166], which is regulated by the expression of TCF4 in CRC cells to promote LM [167].

TAMs play an important role in CRLM. It has been reported that extracellular matrix glycoprotein spondin 2 (SPON2) reshapes the cytoskeleton and activates integrin β1/PYK2 signalling to promote the migration of TAMs, which increases the infiltration of TAMs and promotes the metastasis of CRC [168]. Furthermore, CRC-derived lipids reshape the metabolism of TAMs with the help of CD36, thus inducing TAM M2 polarisation to drive the development of LM [169]. Moreover, CRC-derived exosomal miRNAs can induce M2 polarisation, thus driving the EMT program, which correspondingly promotes the progression and metastasis of CRC [35, 170]. Additionally, the metastasis-related secreted protein Collagen Triple Helix Repeat Containing 1 (CTHRC1) enhances the infiltration of M2-like macrophages to remodel an immunosuppressive TME in the liver [171]. Mechanistically, CRC-derived CTHRC1 interacts with the TGF-β receptor in macrophages to activate TGF-β signalling to promote CRLM.

With the development of single-cell profiling, TAMs have been classified as *C1QC*^+^ TAMs, *SPP1*^+^ TAMs and *MRC1*^+^*CCL18*^+^ TAMs [151, 165, 172]. In a previous study, single-cell analysis showed that *MRC1*^+^*CCL18*^+^ macrophages and *SPP1*^+^ macrophages are the predominant M2 cell subsets in liver metastatic tissue [151]. Consistently, the presence of *SPP1*^+^ macrophages in liver metastatic tissue was reported in a study by Liu et al. Therefore, *SPP1*^+^ macrophages may be a potential culprit in CRLM. Moreover, *MRC1*^+^*CCL18*^+^ macrophages infiltrating liver metastatic tissue exhibit high metabolic activities, thus suggesting that they may promote LM through metabolic pathways [151]. *SPP1*^+^ macrophages are found in mesenteric lymph nodes with metastasis but not in mesenteric lymph nodes without metastasis, thus indicating that *SPP1*^+^ macrophages play a role in facilitating the expansion of disseminated cancer cells [165]. Furthermore, it has been reported that in microsatellite-stable (MSS) CRC, *SPP1*^+^ macrophages and fibroblasts communicate very closely via the ligand‒receptor pathway, which may help to shape an immunosuppressive TME in the liver [173]. However, more studies are required to understand the mechanisms by which different subtypes of macrophages promote LM.

Overall, as the leading tumour-infiltrating immune cells in the TME [174–176], TAMs play a critical role in the progression and metastasis of CRC. Their high proportion is closely related to a worse prognosis [165, 177]. Specific subsets of macrophages, including *MRC1*^+^*CCL18*^+^ and *SPP1*^+^ macrophages, may serve as potential therapeutic targets for CRLM.

#### Myeloid-derived suppressor cells

MDSCs are one of the key contributors to the formation of an immunosuppressive TME in the liver [178]. They mediate immune evasion by inducing the production of T_reg_ cells [179], thus inhibiting NK cell function [180] and impairing the antigen-presenting activity of DCs [181]. In addition, MDSCs facilitate cancer progression and metastasis in a nonimmune manner by producing MMP-9, which is a primary regulator of EMT [182], as well as VEGF, in order to promote TME remodelling and angiogenesis [20]. MDSCs are mainly classified as granulocytic or polymorphonuclear MDSCs (PMN-MDSCs) and monocytic MDSCs (M-MDSCs). The phenotypic and molecular features of these subtypes are difficult to identify [183]. Furthermore, the accumulation of MDSCs is one of the most dominant immunological features of CRC and is associated with disease progression and metastasis [184, 185].

MDSCs facilitate the formation of a PMN and the metastatic colonisation of CRC [186, 187]. Clinically, the high expression of CCL15 in patients with CRC results in the recruitment of more CCR1^+^ MDSCs, which is associated with the loss of SMAD4 (which is a TGFβ-relevant transcription factor) and promotes CRLM [188, 189]. In an orthotopic mouse model of CRC, CXCR2-expressing MDSCs are recruited from the circulatory system to the liver by CXCL1 secreted from CRC cells in the premetastatic liver, which facilitates the growth of disseminated CRC and its metastasis to the liver [187]. Mechanistically, sphingosine-1-phosphate receptor 1 (S1PR1)–STAT3 signalling in CRC cells results in the production of IL-6 to induce the activation of S1PR1 and p-STAT3 in MDSCs, thus leading to the formation of a PMN in the liver to promote CRLM [190]. Zeng et al. reported that the overexpression of hepatocyte-derived cell cycle-related kinase (CCRK) increases CXCL1 production to recruit PMN-MDSCs, thereby impairing NKT cell-mediated immunosurveillance, which dramatically promotes the metastasis of CRC cells to the liver [191]. M-MDSC-produced CCL7 binds to CCR2 on micrometastatic cells and stimulates the JAK/STAT3 pathway to activate dormant cells, thereby promoting the progression of CRLM [192]. Moreover, the inhibition of CCL7 may represent a potential strategy for preventing recurrent CRLM.

However, it is difficult to target MDSCs because they do not have a specific phenotype that differs from other mature granulocytes. Therefore, further research is required to identify therapeutic targets.

#### Natural killer cells

Under physiological conditions, NK cells are enriched in the liver and contribute to defending against infection and eliminating cancer cells [69]. After NK cells encounter cancer cells and are activated, they release PRF and GZM, thus leading to osmotic lysis and apoptosis of cancer cells [193]. Additionally, NK cells can directly kill target cells via the expression of TNF-related apoptosis-inducing ligand and FasL [194]. NK cells function in tumour immunosurveillance and elicit inflammatory responses by producing cytokines and chemokines [195].

NK cells can eliminate disseminated cancer cells to control metastasis [196]. A high proportion of NK cells indicates a good prognosis in patients with CRLM [197]. However, in the highly glycolytic environment of CRLMs, lactic acid causes the apoptosis of NK cells by downregulating their intracellular pH [198]. In addition, MDSCs attenuate the immunoreaction of NK cells by releasing NO, which interferes with FcR-mediated functions of NK cells, such as antibody-dependent cellular cytotoxicity (ADCC) and cytokine generation [199]. In a previous study on murine models of CRLM, compared with conventional NK (cNK) cells, liver-resident natural killer (LrNK) cells had a high expression of RORα, which is required to maintain LrNK cells but has no impact on cNK cells. The conditional knockout of *Rorα* aggravated CRLM, thus indicating that RORα is required for LrNK cell-mediated antitumour immunity. However, the RORα agonist SR1078 restrained CRLM [200]. Clinically, LrNK cells are significantly depleted in CRLM due to the accumulation of tumour-derived lactate, thus resulting in mitochondrial dysfunction and apoptosis of NK cells [198]. The targeting of lactate in the TME may restore the tumour-killing effects of NK cells and benefit patients with CRLM. Altogether, LrNK cells exert a great antitumour impact on CRLM and are closely related to prognosis; therefore, they may be qualified as specific therapeutic targets for CRLM.

#### Dendritic cells

Dendritic cells are the classical APCs that exert considerable influence in triggering antigen-specific immune responses and inducing immune tolerance [201–204]. The antigen-presenting function of conventional DCs (cDCs) is important for the antitumour response of effector T cells [205–207]. Efficient antigen presentation increases the polarisation of CD4^+^ Th1 cells and the activation of CD8^+^ T cells [208, 209].

DCs are heterogeneous and exhibit different characteristics. Compared with plasmacytoid DCs (pDCs), cDCs can more efficiently initiate an immune response against cancer cells [210]. In ICB-treated mouse models of orthotopic pMMR CRLM, the proportion of activated CD8^+^ T cells, CD4^+^ T cells and cDCs is lower in metastatic tumours than in subcutaneous tumours [211]. Liver-derived pDCs have a poor capability to stimulate the proliferation of T cells, thus resulting in anergy of effector T cells and immune suppression to maintain inherent liver tolerogenicity [212, 213]. Moreover, liver-resident regulatory DCs differentiated from bone marrow-derived progenitors secrete high levels of IL-10 but low levels of IL-12, thereby inhibiting effective T-cell function to maintain liver tolerance [214]. A subset of cDCs in CRLM identified as DC3s induces a proinflammatory phenotype and is correlated with a poor prognosis [165]. DC3s may be considered as a promising target for improving the therapeutic outcome of immunotherapy in CRLM. Further investigations are required to elucidate the mechanism by which DC3s promote CRLM.

#### Tumour-associated neutrophils

Similar to TAMs, TANs play a dual role in cancer progression by both promoting and inhibiting the growth and metastasis of cancer [215]. Specifically, TANs facilitate activated T-cell immune reactions by presenting antigens and releasing IL-18 to induce the activation of NK cells [216]. In contrast, TANs release CCL2 and CCL17 to recruit CCR2^+^ M2 macrophages and CCR4^+^ T_reg_ cells, which shape a suppressive TME in the liver, thus promoting the progression and metastasis of cancer [217]. Additionally, TANs produce MMP-9 and neutrophil elastase to promote the extravasation of cancer cells and drive disseminated cancer cells to metastasise [218].

The accumulation of TANs has been demonstrated to be necessary for the formation of an omental PMN in orthotopic ovarian cancer models [219]. Ovarian cancer induces neutrophils to form NETs, which trap ovarian cancer cells and facilitate their implantation on the omentum [219]. Therefore, NETs can promote the metastasis of ovarian cancer. Several in vivo and in vitro studies on CRC have reported that the formation of NETs is enhanced by cancer-derived IL-8. These NETs can trap CRC cells in the liver and promote their invasive and metastatic capabilities [220–222]. In addition, anterior gradient-2 (AGR2) released from TANs can promote metastasis in murine models of CRLM. TAN–CRC cell crosstalk between TAN-derived AGR2 and CRC-derived TGF-β1 is considered the primary driver of CRLM [223]. Collectively, TAN is an effective potential target for the treatment of CRLM. However, further investigations are required to explore and develop TAN-based therapeutic strategies for CRLM.

In conclusion, immune cells involved in CRLM shape the susceptible suppressive immune microenvironment for tumour invasion and metastasis in CRC (Table 2). Immunotherapeutic strategies that can reverse the immunosuppressive microenvironment or strengthen effector immunity may be effective against CRLM.

**Table 2:** Immune cells involved in the liver immune microenvironment of CRLM

Immunological characteristics	Immune cells	Roles in CRLM
Immunosuppressive	T_reg_ cells	Inhibit effective responses of effector T cells and contribute to immune evasion of CRC
	M2 macrophages	Secret cytokines including IL-10, TGF-β, CCL17 and CCL22 to induce the formation of an immunosuppressive TME; attenuate Th1 adaptive immunity; produce MMPs to regulate matrix remodelling
	MDSCs	Induce the production of T_reg_ cells and repress the function of effective NK cells; produce MMP-9 and VEGF to promote TME remodelling and angiogenesis
	TANs	Recruit M2 macrophages and T_reg_ cells to shape a suppressive TME in the liver; produce MMP-9 and neutrophil elastase to promote extravasation of CRC cells; form NETs to trap and facilitate the implantation of CRC cells to the liver; produce AGR2 to communicate with CRC cells to drive CRLM
Immuno-effective	CD4^+^ Th1 cells	Produce IFNγ and TNF-α, leading to cell-mediated killing
	CD4^+^ Th2 cells	Secrete IL-4, which assists in the activation of humoral immunity
	CD8^+^ T cells	Secret cytotoxic granules and release proteins to kill disseminated CRC cells; produce TNF-α, IL-2 and IFNγ to strengthen the cytotoxicity
	M1 macrophages	Kill cancer cells directly by releasing cytotoxic reactive oxygen species (ROS), NO and IL-12
	NKs	Release IFNγ and NO toupregulate the expression of functional Fas in CRC cells; exert cytotoxic effects to eliminate CRC cells by PRF and GZM and kill CRC cells directly by releasing the apoptosis-inducing ligand and FasL
	DCs	Present antigens and deliver co-stimulatory signals for T-cell activation to initiate effective immune responses
	TANs	Activate T-cell immune reaction by presenting antigens; release IL-18 to induce the activation of NK cells

*PRF *Perforin, *GZM *Granzyme, *MMP *Matrix metalloproteinase, *AGR2 *Anterior gradient-2

### Extracellular vesicles in the immune microenvironment of CRLM

**Fig. 4:**
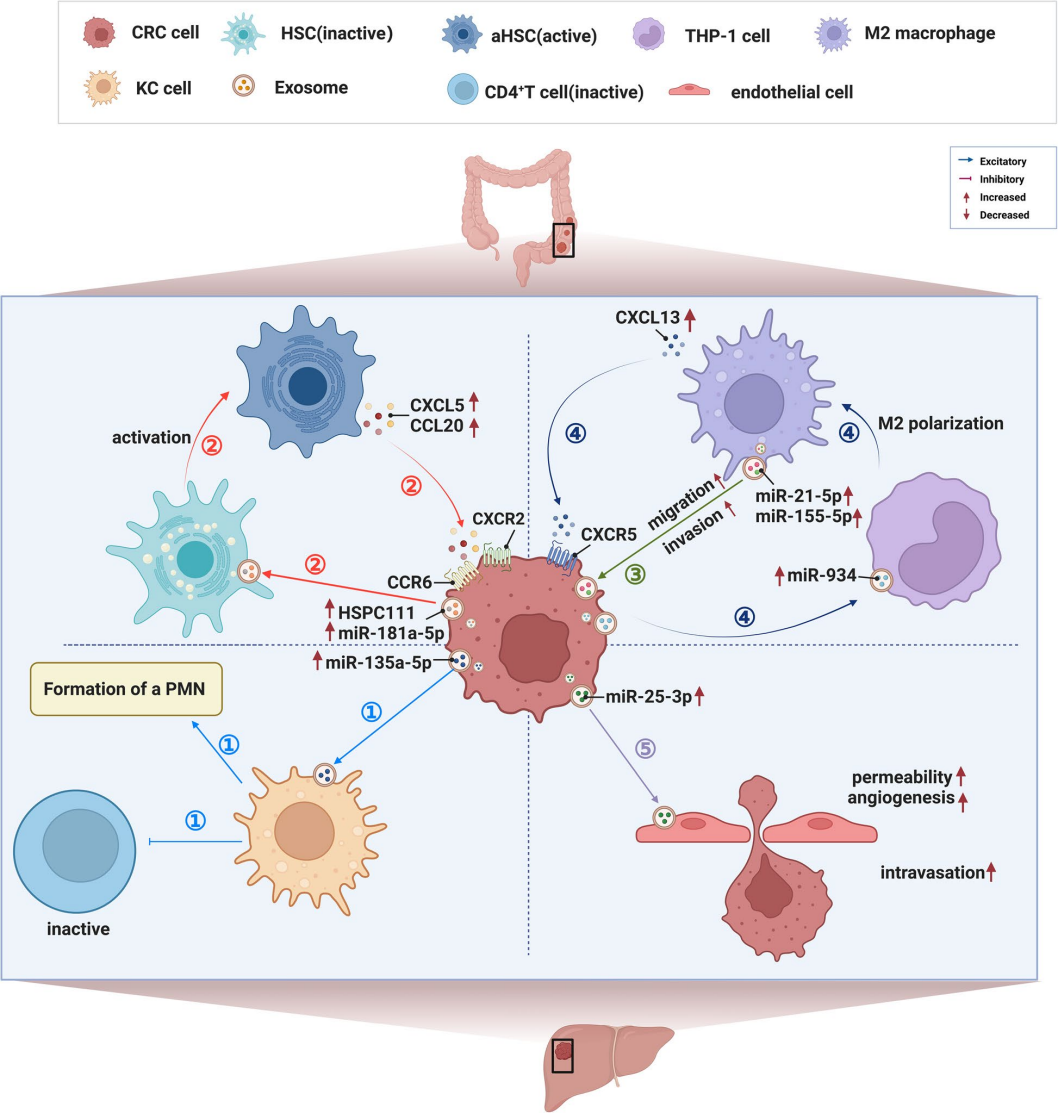
Schematic diagram depicting extracellular vesicles in the immune microenvironment of CRLM. Various cells in the hepatic immune microenvironment interact with CRC cells via extracellular vesicles to form a sophisticated immunosuppressive microenvironment that contributes to CRLM. The different pathways are indicated by different coloured arrows. ① CRC-derived hypoxia-induced exosomal miR-135a-5p is phagocytosed by KCs, thus blocking CD30-mediated CD4^+^ T-cell activation and promoting cell adhesion. ② Highly mCRC cells produce EV-packaged miR-181a-5p that activates HSCs. aHSCs release CCL20, which interacts with CCR6 expressed on CRC cells and activates CRC cells to promote the release of EV-packaged miR-181a-5p, thus contributing to reshaping the hepatic TME and forming a PMN; CRC-derived exosomal HSPC111 promotes the activation of HSCs, thus leading to the upregulation of CXCL5, which targets CRC-expressed CXCR2, increases the secretion of exosomal HSPC111 from CRC cells and promotes CRLM. ③ M2 macrophages release exosomal miR-21-5p and miR-155-5p, after which they shuttle into CRC cells, which contributes to the migration and invasion of CRC. ④ Exosomal miR-934 secreted from CRC cells induces M2 macrophage polarisation to promote CRLM. M2 macrophages release CXCL13, which interacts with CXCR5 in CRC cells and promotes the transcription of miR-934. ⑤ CRC cells secrete exosomal miR-25-3p to stimulate endothelial cells, thus leading to vascular leakage and vasculogenesis

EVs refer to various nanosized vesicles with membrane structures released by cells [224, 225]. According to their diameter and the mechanisms of biogenesis, they are classified into three subgroups (exosomes, microvesicles and apoptotic bodies). Exosomes have attracted substantial interest and have been widely investigated in recent years [226]. EVs carry bioactive molecules such as nucleic acids, proteins and lipids for intercellular delivery and facilitate intercellular communication [227, 228]. They are an important aspect of the immune microenvironment. CRC-derived EVs in the immune microenvironment facilitate the relocation and aggression of CRC, which contributes to LM [229–232]. In previous studies, we elucidated the molecular mechanisms underlying the involvement of EVs in the formation of a hepatic PMN and the metastasis of CRC, and we also identified promising functional biomarkers for CRC [35, 112, 233, 234], thus indicating that EVs may serve as a therapeutic target and a prognostic and diagnostic biomarker for CRLM (Fig. 4).

Several studies have validated the pivotal role of EVs in the formation of a hepatic PMN. After being phagocytosed by KCs, CRC-derived EV-packaged miR-135a-5p can inhibit CD30-induced activation of CD4^+^ T cells and promote cell adhesion, which facilitates the development of a PMN for CRLM [101]. In a previous study, we reported that EV-packaged miR-181a-5p secreted by CRC cells activates HSCs. The interaction between CCL20 released from aHSCs and CCR6 expressed on CRC cells activates CRC cells to promote the release of exosomal miR-181a-5p, which generates a positive feedback loop to reshape the hepatic TME and form a PMN [112]. In mouse models of CRLM, endothelial cells stimulated by CRC-derived exosomal miR-25-3p can lead to vascular leakage and vasculogenesis, thus contributing to the formation of a PMN and enhancing CRLM [235].

EVs play an important role in intercellular communication, such as between CRC cells and other cells, in CRLM. M2 macrophage-derived exosomes can deliver miR-21-5p and miR-155-5p to CRC cells. Additionally, miR-21-5p and miR-155-5p are internalised by CRC cells and targeted to the BRG1 coding sequence, thus leading to a decrease in the expression of BRG1 in CRC cells and contributing to the migration and invasion of CRC [236]. In a previous study, we demonstrated that exosomal miR-934 in the immune microenvironment induces M2 macrophage polarisation to promote CRLM. Additionally, CXCL13 released by M2 macrophages interacts with CXCR5 on CRC cells to promote the transcription of miR-934 [35]. CRC-derived exosomal HSPC111 can promote the activation of HSCs, thus leading to the upregulation of CXCL5, which targets CXCR2 expressed on CRC cells, increases the release of exosomal HSPC111 from CRC cells and promotes CRLM [237].

Altogether, as coordinators of intercellular communication in the dynamic network of the TME, EVs are responsible for the progression and metastasis of cancer [238] and can serve as noninvasive markers for the screening and management of CRLM [239, 240]. A better understanding of the regulatory mechanisms of EVs can help to generate antitumour responses and design efficient EV-based diagnostic and therapeutic strategies for CRLM.

## Therapies for CRLM

### Surgical resection

At present, surgical resection is an effective therapeutic option for resectable CRLM [241]. The two commonly used surgical strategies include simultaneous resection and delayed resection. The results of existing studies on the selection of simultaneous or delayed resection are inconsistent [242–244]. The 5-year survival rate of patients with CRLM after resection can be improved to 50% [241]. However, only 10–30% of patients with localised LM are eligible for resection after diagnosis [245]. Moreover, 52% of patients develop postoperative recurrence of CRLM [246], thus resulting in a high mortality rate. Therefore, there is a need to explore novel therapeutic modalities for CRLM.

### Systemic and conversion therapies

Systemic therapy is a more favourable treatment option for nonresectable CRLM. In addition to improving the quality of life and prolonging survival, effective systemic therapy can transform unresectable lesions into resectable lesions, which is known as conversion therapy [247]. According to the guidelines recommended by the National Comprehensive Cancer Network (NCCN) [248], first-line chemotherapy regimens for patients eligible for intensive therapy are FOLFOX (5-fluorouracil combined with leucovorin plus oxaliplatin), CAPEOX (combination of capecitabine and oxaliplatin), FOLFIRI (5-fluorouracil plus leucovorin and irinotecan) and injectable 5-fluorouracil/leucovorin or capecitabine. The use of FOLFOXIRI (5-fluorouracil combined with leucovorin, oxaliplatin and irinotecan) in conversion therapy may maximise tumour shrinkage and improve the eventual outcomes of surgery in patients with potentially resectable CRLM. Effective conversion therapy can allow for 12.5% of patients with unresectable CRLM to undergo liver resection, thus resulting in improved survival rates [249]. However, several adverse effects are associated with this regimen, which should be carefully considered.

Chemotherapy combined with targeted therapy can yield a better outcome for patients who are tolerant to aggressive therapy [250]. Drugs targeting epithelial growth factor receptor (EGFR) and VEGF are commonly used in combination with chemotherapeutic drugs. A phase III trial demonstrated that cetuximab and panitumumab (which are monoclonal antibodies against EGFR) can suppress the downstream signalling pathways of EGFR to effectively inhibit disease progression and provide clinical benefits to patients with mCRC [251]. However, cetuximab and panitumumab are only indicated in patients with wild-type RAS/BRAF [248]. Bevacizumab targets VEGF and plays a significant role in antiangiogenesis [252]. Several clinical studies have demonstrated that compared with independent chemotherapy, the combination of bevacizumab and chemotherapy improves progression-free survival (PFS) and overall survival (OS) [253–255]. However, the effectiveness of antiangiogenic therapy varies among patients, with some patients failing to benefit from this therapy and others developing tolerance or worse, aggressive, metastatic and other adverse outcomes [256, 257]. Therefore, more effective treatment strategies are urgently needed.

### Immunotherapy targeting the immune microenvironment to eliminate immunosuppression

#### Immune checkpoint blockade

At present, the direct blockage of immune checkpoints to inhibit immune escape is the most well-established immunotherapeutic approach that has demonstrated excellent efficacy in the treatment of several cancers [258–265]. However, unlike the response of patients with other cancers, that of patients with CRC to immune checkpoint blockade (ICB) depends on the DNA microsatellite instability (MSI) or mismatch repair (MMR) status [266–268]. ICB agents are effective in patients with mCRC but mostly in those patients with high microsatellite instability (MSI-H) or deficient mismatch repair (dMMR) [269–271]. CRC with MSI-H/dMMR has a high tumour mutational burden that induces tumour-specific neoantigens to alert immune cells and subsequently recruits numerous T cells, which improve sensitivity to ICB [272, 273].

The phase II CheckMate 142 trial demonstrated that patients with mCRC with MSI-H/dMMR who were pretreated with nivolumab, which is a PD-1 immune checkpoint inhibitor, had an objective response rate (ORR) of 31.1% and 12-month PFS and OS rates of 50% and 73%, respectively [270]. Additionally, a subsequent study reported that patients with mCRC with MSI-H/dMMR responded to the combination of nivolumab plus ipilimumab (a CTLA-4 inhibitor) as second-line therapy with a higher ORR of 55%, and the clinical benefit of the combination therapy was better than that of nivolumab monotherapy [274]. A recent investigation showed that the combination of nivolumab and low-dose ipilimumab, which was a first-line treatment, was well tolerated by patients with mCRC with MSI-H/dMMR and provided durable and robust clinical benefit characterised by an ORR of 69% [275], which indicated that ICB agents may serve as new and safe first-line drugs for the treatment of mCRC. The KEYNOTE-164 study showed that pembrolizumab, which is a PD-1 inhibitor, was an effective and safe ICB agent with an ORR of 33% in patients with treatment-refractory mCRC with MSI-H/dMMR [276]. As the first-line standard treatment, pembrolizumab outperforms chemotherapy in terms of PFS (16.5 months versus 8.2 months, respectively) in patients with mCRC with MSI-H/dMMR [277].

However, ICB alone or in combination shows weak outcomes in patients with MSS/mismatch repair-proficient (pMMR) mCRC. A phase II study demonstrated that lenvatinib combined with pembrolizumab resulted in a poor median PFS of 2.3 months with a 50% incidence of treatment-related adverse events in non-MSI-H/pMMR mCRC [278]. In the METIMMOX study, with short-course sequential oxaliplatin-based chemotherapy (FLOX), the addition of nivolumab treatment prolonged PFS by only 1 month compared with chemotherapy alone [279]; however, the therapeutic strategy was not as effective in patients with MSI-H/dMMR CRC. Another study demonstrated that regorafenib plus nivolumab resulted in an encouraging ORR of 21.7% in patients with MSS/pMMR CRC without LM; however, the ORR was remarkably lower in patients with LM [280]. Therefore, ICB treatment of patients with MSS/pMMR CRLM is challenging.

Overall, patients with MSI-H/dMMR CRC exhibited a positive response to ICB, whereas patients with MSS/pMMR CRC exhibited almost no response to ICB (Table 3). As one of the most promising antitumour treatments, ICB-based therapy has great potential for the treatment of CRC. Further investigations are required to verify the efficiency and safety of ICB agents in patients with CRLM, irrespective of their MSI or MMR status.

**Table 3:** Main immune checkpoint blockade agents for the treatment of metastatic colorectal cancer

Intervention	Key trial (NCT number)	Design (N)	Subject	Main results
Nivolumab	CheckMate 142 (NCT02060188)	Phase II (*N* = 74)	MSI-H/dMMR mCRC	PFS rate: 50% (12 m) OS rate: 73% (12 m) ORR: 31.1% Grade ≥ 3 TRAEs: 21%
Nivolumab + ipilimumab	CheckMate 142 (NCT02060188)	Phase II (*N* = 119)	MSI-H/dMMR mCRC	PFS rate: 71% (12 m) OS rate: 85% (12 m) ORR: 55% Grade ≥ 3 TRAEs: 32%
Nivolumab + ipilimumab	CheckMate 142 (NCT04008030)	Phase II (*N* = 45)	MSI-H/dMMR mCRC	PFS rate: 76.4% (12 m) OS rate: 84.1% (12 m) ORR: 69% Grade ≥ 3 TRAEs: 22%
Pembrolizumab	KEYNOTE-164 (NCT02460198)	Phase II (*N* = 124, 61 in cohort A and 63 in cohort B)	MSI-H/dMMR mCRC	PFS: 2.3 m in cohort A and 4.1 m in cohort B OS: 31.4 m in cohort A and not reached in cohort B ORR: 33% in cohort A and 33% in cohort B Grade ≥ 3 TRAEs: 16% in cohort A and 13% in cohort B
Pembrolizumab	KEYNOTE-177 (NCT02563002)	Phase III (*N* = 307)	MSI-H/dMMR mCRC	PFS: 8.2 m with chemotherapy and 16.5 m with pembrolizumab treatment Estimated restricted mean survival: 10.8 m with chemotherapy and 13.7 m with pembrolizumab treatment ORR: 33.1% with chemotherapy and 43.8% with pembrolizumab treatment Grade ≥ 3 TRAEs: 66% with chemotherapy and 22% with pembrolizumab treatment
Pembrolizumab + lenvatinib	LEAP-005 (NCT03797326)	Phase II (*N* = 32)	Non-MSI-H/pMMR mCRC	Median PFS: 2.3 m Median OS: 7.5 m ORR: 22% Grade ≥ 3 TRAEs: 50%
Nivolumab + FLOX	METIMMOX (NCT03388190)	Phase II (*N* = 54)	MSS mCRC	PFS: 5.6 m with FLOX alone and 6.6 m with repeat sequential FLOX and nivolumab Complete response: 0% with FLOX alone and 16% with repeat sequential FLOX and nivolumab Ongoing objective response: 23% with FLOX alone and 32% with repeat sequential FLOX and nivolumab
Nivolumab + regorafenib	NCT04126733	Phase II (*N* = 70)	MSS/pMMR CRC	Median PFS: 15 w in patients without LM and 8 w in patients with LM Median OS: 52 w in patients without LM and 47 w in patients with LM ORR: 21.7% in patients without LM and 0% in patients with LM Grade 3–4 TRAEs: 63%

*mCRC *metastatic colorectal cancer, *PFS *Progression-free survival, *OS *Overall survival, *ORR *Objective response rate, *TRAEs *Treatment-related adverse events, *m *months, *w *weeks

#### Adoptive cell therapy

In adoptive cell therapy, cells with antitumour activity in vivo are isolated, modified and cultured in vitro and infused back into patients for antitumour treatment [281]. As an important branch of immunotherapy, adoptive cell therapy has made significant contributions to advancing the development of immunotherapy [281, 282]. In particular, chimeric antigen receptor T-cell (CAR-T) therapy has demonstrated successful results in the treatment of haematological malignancies and is the only cell product approved by the FDA [283, 284]. Given the effectiveness of CAR-T therapy, new adoptive cell therapies have been developed for the treatment of solid tumours [285]. In CRC, CAR-T therapy remains a major focus of research and has a distinct advantage over other cell therapies (Table 4).

**Table 4:** CAR-T therapy for metastatic colorectal cancer in preclinical or clinical trials

Intervention	Stage of research	Trial ID	Design (N)	Subject
Anti-CEA CAR-T cells	Clinical trial	NCT02349724	Phase I (*N* = 10)	Relapsed and refractory mCRC
Human GUCY2C-targeted murine CAR-T cells	Preclinical trial	-	-	-
CYAD-01	Clinical trial	NCT03310008	Phase I (*N* = 36)	CRLM
CYAD-101	Clinical trial	NCT03692429	Phase I (*N* = 49)	Unresectable mCRC
CYAD-101 + pembrolizumab	Clinical trial	NCT04991948	Phase I (*N* = 34)	Unresectable mCRC

*mCRC *metastatic colorectal cancer

In a small-sample phase I clinical study on patients with CEA^+^ mCRC treated with gradient doses of CAR-T cells, 70% of participants regressed from progressive disease to stable disease without experiencing severe adverse events [286]. Therefore, CAR-T therapy is a safe and hopeful immunotherapeutic option for the effective management of mCRC. A preclinical study demonstrated that human guanylyl cyclase C (GUCY2C)-targeted CAR-T cells triggered T-cell activation, exerted antitumour effects and alleviated CRLM in both syngeneic and human CRC xenograft murine models [287]. Due to the fact that the safety of GUCY2C-targeted CAR-T cells has only been examined in murine models, these cells should be cautiously used in humans because GUCY2C is selectively expressed in intestinal epithelial cells. Furthermore, CYAD-01 (which is an autologous CAR based on natural killer group 2D [NKG2D] with a single generic construct) induces both innate and adaptive immunity to regulate the immunosuppressive TME, thus presenting novel insights into CAR-T-cell therapy [288]. Moreover, the phase I SHRINK trial showed that CYAD-01 in combination with standard chemotherapy elicited partial response (PR) in 25% of participants with resectable mCRC (*n* = 4) and reduced tumour burden in 60% of participants with refractory mCRC (*n* = 5) without inducing cumulative toxicity [289].

In the ALLOSHRINK trial, combination therapy with CYAD-101 (a nongene-edited, allogeneic, second-generation NKG2D CAR-T-cell product) and chemotherapy was well tolerated with no evidence of graft-versus-host disease and was reported to be a prospective treatment for patients with incurable mCRC who relapsed after multiple lines of therapy [290]. The KEYNOTE-B79 phase 1b multicentre clinical study was open and recruited patients to assess the effectiveness of CYAD-101 plus pembrolizumab in refractory mCRC [291]. Therefore, CYAD-01 and CYAD-101 CAR-T therapies are novel immunotherapeutic strategies for the effective treatment of mCRC.

In conclusion, appropriate CAR targets are poorly expressed in normal cells but are enriched in cancer cells. Therefore, CAR-T cells can precisely target cancer cells and improve the survival of patients with mCRC. Further preclinical/clinical trials should be conducted to demonstrate the actual effectiveness of CAR-T-cell therapy in solid tumours, including CRC.

#### Cancer vaccines

Cancer vaccines are prospective therapeutic options in cancer immunotherapy [292–294]. Unlike ICB and adoptive cell therapy, cancer vaccines exert antitumour effects by introducing tumour antigens into the body, activating immune responses and using the active immune function to kill cancer cells [295, 296]. At present, no products have been approved for developing cancer vaccines against CRC; however, relevant clinical trials are actively ongoing (Table 5).

**Table 5:** Clinical trials targeting the immune landscape for the treatment of metastatic colorectal cancer

Type of cancer vaccine	Tumour-associated antigen	Trial ID	Phase	Subject
Cancer cell vaccine	Autologous cancer cells	NCT00016133	Phase I/II	Stage II or III colon cancer
Cancer cell vaccine	Autologous cancer cells	NCT02448173	Phase III	Stage II colon cancer
DC vaccine	Mutated peptides	NCT03730948	Phase I	Stage I and II hypermutated colorectal cancer
DC vaccine	Autologous tumour homogenate	NCT02919644	Phase II	Stage IV colorectal cancer
Peptide vaccine	GUCY2C–PADRE	NCT01972737	Phase I	Stage I or stage II colon cancer
Peptide vaccine	12 unique epitopes	NCT03391232	Phase I	mCRC

*mCRC *metastatic colorectal cancer

Various vaccine strategies have been designed, including whole tumour cell-based, protein- or peptide-based and DC-based vaccines [297–299]. In a randomised clinical trial, patients with colon cancer who received an autologous cancer cell-based vaccine had a significantly longer recurrence-free interval (*p* = 0.011) and recurrence-free survival (*p* = 0.032); however, disease-specific survival and OS showed no improvement [300]. Based on this foundation, further clinical studies (Clinicaltrials.gov Identifier: NCT00016133, NCT02448173) have been conducted to examine the protective effects of the autologous cancer vaccine against tumour recurrence after colon cancer surgery [301]. DC-based vaccines are powerful contributors to antigen presentation and the initiation of antitumour immunity [302–304]; however, the development of DC-based vaccines for CRC is currently at an early stage. In a preclinical murine model, effective immunotherapy using tumour-associated antigen-loaded cDC-based vaccines increased the infiltration of activated effector T cells and inhibited tumour growth [305]. Two related clinical trials (Clinicaltrials.gov Identifier: NCT03730948, NCT02919644) on DC-based vaccines are ongoing to develop strategies for preventing the progression of surgically resected stage I and II hypermutated CRC or curatively resected stage IV CRC. Moreover, peptide-based vaccines have strong specificity and can easily elicit an effective immune response [306, 307]. In a phase I clinical trial, the adenovirus (Ad5)-GUCY2C-PADRE vaccine was efficient in patients with CRC and did not cause grade-3/4 toxic events during the 6-month follow-up period after vaccination [308]. Another phase I study demonstrated that the combination of a single-dose PolyPEPI1018 vaccine and maintenance therapy with fluoropyrimidine and bevacizumab was strongly effective, with 96% of vaccine peptides inducing T-cell responses without causing grade 3 or higher adverse events in patients with mCRC [309].

Based on these encouraging results, cancer vaccines may represent an immunotherapeutic strategy that is not limited by the DNA MSI or MMR status. Cancer vaccines can stimulate immune surveillance to combat initially undetected microscopic lesions and consequently enhance the survival of patients with CRLM.

**Fig. 5:**
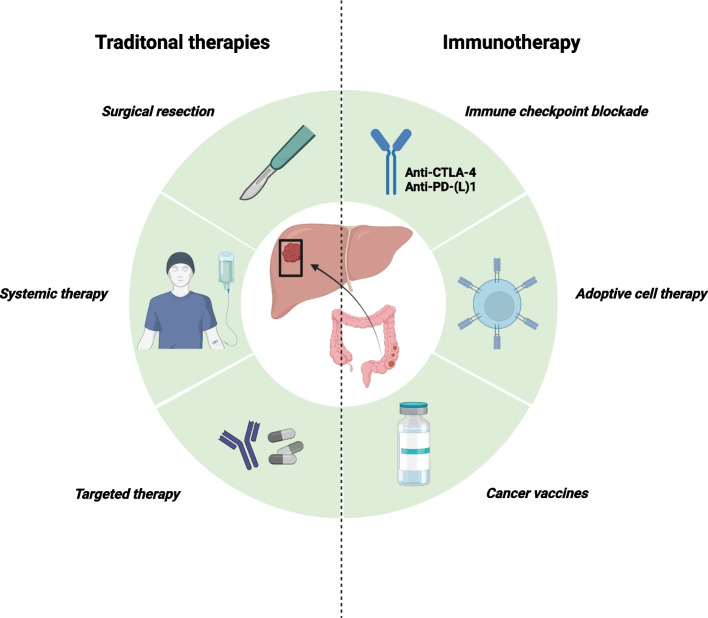
Therapies for CRLM

There are multiple therapeutic options for CRLM; however, they fail to meet the requirements for a disease-free prognosis (Fig. 5). Immunotherapy is an emerging and effective weapon in the fight against CRLM that requires further research to explore its superior potential value.

## Discussion and perspectives

Liver metastasis is the most common site of metastasis in CRC and is the leading cause of death in patients with CRC. The liver is a characteristic immune-tolerant organ in which resident liver cells, recruited inflammatory and immune cells and active protein molecules interact with each other; additionally, EVs act as important mediators of intercellular communication. The intricate characteristics and mechanisms of the hepatic immune microenvironment that directly or indirectly induce immunosuppression and contribute to the regulation of cancer metastasis should be extensively investigated to explore potential therapeutic targets for CRLM.

The formation of a PMN in CRC is an important prerequisite for LM, which is a progressive process that triggers local changes such as vascular leakage, ECM remodelling and systemic effects on the immune system. Induced by the combined systemic effects of CRC-secreted factors and EVs, the PMN shapes a microenvironment that is favourable for LM, which makes the distant liver a favourable site for the colonisation of disseminated cancer cells. However, the underlying mechanisms responsible for the formation of the PMN remain uncertain and warrant further investigations.

ICB-based treatment is only effective in patients with MSI-H/dMMR CRC but not in patients with MSS/pMMR CRC. Moreover, another challenge involves the potentially deleterious side effect known as hyperprogression that occurs in some patients after ICB therapy and is independently associated with advanced age and higher metastatic load. With the increasing use of ICB therapy in clinical practice, more studies are required to elucidate the potential mechanisms and to identify the predictors of hyperprogression, which would allow for patients at high risk for life-threatening immune-related adverse events to be screened before ICB therapy. As emerging immunotherapeutic strategies, CAR-T-cell therapy and cancer vaccines may revolutionise the era of cancer immunotherapy. Despite the excellent efficacy of CAR-T-cell therapy in haematological malignancies, its use in solid tumours may be limited due to the trafficking barriers of CAR-T cells, their weak ability to infiltrate solid tumours and their off-target effects. Furthermore, tumour vaccines are a type of individualised immunotherapy with high specificity and few side effects. However, the effective translation of cancer vaccines in clinical practice remains challenging. Further preclinical and clinical trials should be conducted to demonstrate the efficacy of immunotherapy in mCRC and to identify novel therapeutic targets or to develop combination strategies to improve or activate antitumour immune responses for the effective treatment of CRLM.

## Data Availability

Not applicable.

## Abbreviations

CRC: Colorectal cancer
LM: Liver metastasis
CRLM: Colorectal liver metastasis
TME: Tumor microenvironment
ECM: Extracellular matrix
EV: Extracellular vesicle
T_reg_: Regulatory T cell
MDSC: Myeloid-derived suppressor cell
TAM: Tumor associated macrophage
APC: Antigen-presenting cell
LSEC: Liver sinusoidal endothelial cell
KC: Kupffer cell
HSC: Hepatic stellate cell
DC: Dendritic cell
NK: Natural killer cell
MHC: Major histocompatibility complex
PD-L1: Programmed cell death 1 ligand 1
PD-1: Programmed cell death-1
PRF: Perforin
GZM: Granzyme
IFNγ: Interferon-γ
CEA: Carcinoembryonic antigen
mCRC: Metastatic CRC
TNF-α: Tumor necrosis factor-α
IL-1α: Interleukin-1α
VEGF: Vascular endothelial growth factor
MMP: Matrix metalloproteinase
CTLA-4: Cytotoxic T lymphocyte antigen-4
A_2A_R: A_2A_ receptor
EMT: Epithelial-mesenchymal transition
CTL: Cytotoxic T lymphocyte
ADCC: Antibody-dependent cellular cytotoxicity
PMN-MDSCs: Polymorphonuclear MDSCs
M-MDSCs: Monocytic MDSCs
S1PR1: Sphingosine-1-phosphate receptor 1
cNK: Conventional NK cell
LrNK: Liver-resident natural killer cell
cDC: Conventional DC
pDC: Plasmacytoid DC
TAN: Tumor-associated neutrophil
NET: Neutrophil extracellular trap
EGFR: Epithelial growth factor receptor
PFS: Progression-free survival
OS: Overall survival
ICB: Immune checkpoint blockade
MSI: Microsatellite instability
MSS: Microsatellite stable
MMR: Mismatch repair
ORR: Objective response rate
CAR-T: Chimeric antigen receptor T cell
GUCY2C: Guanylyl cyclase C
NKG2D: Natural killer group 2D
